# Insights and therapeutic advances in pancreatic cancer: the role of electron microscopy in decoding the tumor microenvironment

**DOI:** 10.3389/fcell.2024.1460544

**Published:** 2024-12-18

**Authors:** Hong Dai, Xingxuan Chen, Jiawen Yang, Yuying Wang, Rodrigo Azevedo Loiola, Aiping Lu, Kenneth C. P. Cheung

**Affiliations:** ^1^ Department of Chemistry, The Hong Kong University of Science and Technology, Kowloon, Hong Kong SAR, China; ^2^ Phenome Research Center, School of Chinese Medicine, Hong Kong Baptist University, Kowloon, Hong Kong SAR, China; ^3^ School of Life Science, Southern University of Science and Technology, Shenzhen, Guangdong, China; ^4^ Oroxcell, Parc Biocitech, Romainville, France

**Keywords:** pancreatic cancer, tumor microenvironment, EM, antibody-drug conjugates, aptamer-drug conjugates

## Abstract

Pancreatic cancer is one of the most lethal cancers, with a 5-year overall survival rate of less than 10%. Despite the development of novel therapies in recent decades, current chemotherapeutic strategies offer limited clinical benefits due to the high heterogeneity and desmoplastic tumor microenvironment (TME) of pancreatic cancer as well as inefficient drug penetration. Antibody- and nucleic acid-based targeting therapies have emerged as strong contenders in pancreatic cancer drug discovery. Numerous studies have shown that these strategies can significantly enhance drug accumulation in tumors while reducing systemic toxicity. Additionally, electron microscopy (EM) has been a critical tool for high-resolution analysis of the TME, providing insights into the ultrastructural changes associated with pancreatic cancer progression and treatment responses. This review traces the current and technological advances in EM, particularly the development of ultramicrotomy and improvements in sample preparation that have facilitated the detailed visualization of cellular and extracellular components of the TME. This review highlights the contribution of EM in assessing the efficacy of therapeutic agents, from revealing apoptotic changes to characterizing the effects of novel compounds like ionophore antibiotic gramicidin A on cellular ultrastructures. Moreover, the review delves into the potential of EM in studying the interactions between the tumor microbiome and cancer cell migration, as well as in aiding the development of targeted therapies like antibody-drug conjugates (ADCs) and aptamer-drug conjugates (ApDCs).

## 1 Introduction

Pancreatic cancer, also known as pancreatic adenocarcinoma, is one of the deadliest malignancies with poor outcomes and pancreatic ductal adenocarcinoma (PDAC) takes up 90% among all the types of pancreatic cancer (Hidalgo, 2010). The overall 5-year survival rate for pancreatic cancer is only 9% in 2020, which is supposed to be the second leading cause of cancer-related mortality in the next decade (Siegel et al., 2020). The low survival rate could mainly attribute to the difficult detection of patients with developing pancreatic cancer which usually leads to diagnosis of the advanced stage when patients developed obvious symptoms and also a shortage of efficient drug administrations for patients despite of various emerging novel therapies (Kleeff et al., 2016; Leroux and Konstantinidou, 2021). For now, the potential cure of early stage pancreatic cancer only can be achieved by the combination of surgical resection and chemotherapy as adjuvants to improve the survival rates (McGuigan et al., 2018). The standard clinical chemotherapy of pancreatic cancer are mainly on the basis of nucleoside analogs including gemcitabine, capecitabine and supplemented agents such as nab-paclitaxel (Kleeff et al., 2016). Although tumor screening, early detection and novel therapies such as CAR-T and immunotherapy have made large progress on breast and colorectal cancers with major improvements in survival durations, little improvements have not shown up within pancreatic cancer (Nevala-Plagemann et al., 2020). Intertumoral heterogeneity, desmoplastic tumor microenvironment and drug resistance are the major reasons heavily hindering the development of drug discovery against pancreatic cancer, which mainly prevent the efficient drug accumulation in pancreatic tumors (Sun et al., 2020).

In recent decades, antibody-drug conjugates (ADCs) have made encouraging progress since the first approval of gemtuzumab ozogamicin (Mylotarg^®^) for the treatment of CD33-positive acute myeloid leukaemia (AML) patients (Lambert and Morris, 2017). Trastuzumab-SMCC-DM1 (Phillips et al., 2008) is also one representative example of ADCs for the treatment of HER2-positive metastatic breast cancer (mBC). Currently, there are 11 ADCs approved by FDA (Table 1) covering indications such as CD33-positive acute myeloid leukaemia, HER2-positive metastatic breast cancer, locally advanced or metastatic urothelial cancer, large B-cell lymphoma (Drago et al., 2021; Khongorzul et al., 2020). However, the effect of ADC against pancreatic cancer is required to be further evaluated although there are indeed some ongoing clinical trials and the adverse effects such as immunogenicity remain to be tackled (Merlino et al., 2019). Oligonucleotides drugs are another novel therapeutics and there are 10 oligonucleotide drugs which have been approved by FDA. As mutated genes such as KRAS and P53 are commonly observed in pancreatic cancer, gene silencing strategy induced by oligonucleotide drugs could be the promising therapy for the treatment of pancreatic cancer but remain challenging owing to the delivery obstacles (Hu et al., 2020). However, up to date, targeting the most commonly mutated genes KRAS and P53 in pancreatic cancer has not yet produced one promising clinical therapeutic (Thomas and Radhakrishnan, 2019), which is mainly because of the targeted delivery problems. Aptamer is one unique form of oligonucleotides and could bind to the molecular targets to exert potential effects just like antibody. Aptamer-drug conjugate is another kind of forms similar to ADCs, many preclinical evidences have also shown the promising results which might be another promising therapy against pancreatic cancer (Li et al., 2017).

**TABLE 1:** ADCs approved by the FDA (Liu et al., 2024).

No.	Product name	Common name	Target	Antibody	Indications	Drug structure
1	Mylotarg	Gemtuzumab ozogamicin	CD33	IgG4	AML (acute myeloid leukemia)	Humanized monoclonal antibody targeting CD33 + a cytotoxic N-acetyl-γ-calicheamicin linked by cleavable hydrazine bonds
2	Adcetris	Brentuximab vedotin	CD30	IgG1	Hodgkin’s lymphoma (HL) and anaplastic large cell lymphoma (ALCL)	Brentuximab (an IgG1 chimeric monoclonal antibody targeting CD30), maleimide-linked fraction (a cleavable dipeptide linkage, mc-VC-PABC) and monomethylauristatin E (MMAE)
3	Kadcyla (T-MD-1)	Trastuzumab emtansine	Her2	IgG1	HER2-positive cancer patients	Formed by linking the drug trastuzumab to emtansine (also known as DM1) through a thioether linker
4	Besponsa	Inotuzumab ozogamicin	CD22	IgG1	Acute lymphoblastic leukemia (ALL)	Coupling of human IgG4 monoclonal antibody targeting CD22 with the cytotoxic chemotherapeutic agent calicheamicin via acid-mediated unstable splicing
5	Lumoxiti	Moxetumomab pasudotox	CD22	IgG1	Adult patients with relapsed/refractory hairy cell leukemia (HCL) who have failed to respond to at least two systemic therapies, including purine nucleoside analogs	Mocilimumab targeting CD22, a 38 kDa fragment of *Pseudomonas* exotoxin a, and linker mc-VC-PABC
6	Polivy	Polatuzumab vedotin	CD79b	IgG1	Adult patients with relapsed/refractory diffuse large B-cell lymphoma (DLBCL)	Antibody CD79b consists of a cleavable dipeptide linkage (mc-VC-PABC) to MMAE
7	Padcev	Enfortumab vedotin	Nectin-4	IgG1	Locally advanced or metastatic uroepithelial cancer (la/ mUC) global treatment	Binding of anti-Nectin-4 antibodies to the cytotoxic drug MMAE by cleavable junctions
8	Enhertu	Trastuzumab deruxtecan	Her2	IgG1	Patients with unresectable or metastatic her2-positive breast cancer	Trastuzumab in combination with exatecan derivatives (topoisomerase I inhibitors)
9	Trodelvy	Sacituzumab govitecan	Trop2	IgG1	Adult patients with metastatic triple-negative breast cancer (TNBC)	Includes an anti-trop-2 antibody, an SN-38 drug load (an active metabolite of irinotecan irinotecan), and a CL2A chemical linkage
10	Blenrep	Belantamab mafodotin	BCMA	IgG1	As a monotherapy for the treatment of adult patients with relapsed or refractory multiple myeloma (R/R MM) who have received at least 4 prior therapies and whose disease is refractory to at least one proteasome inhibitor/immunomodulator/CD38 monoclonal antibody, and whose disease progression was demonstrated on their last therapy	An antibody-drug coupling (ADC) targeting B-cell maturation antigen (BCMA), IgG1 antibody, consists of a microtubule inhibitor, MMAF, linked via a linker resistant to protease degradation
11	Zynlonta	Loncastuximab tesirin	CD19	IgG1	Relapsed/refractory LBCL on second-line or higher systemic therapy, including DLBCL unspecified, DLBCL due to low-grade lymphoma, and high-grade b-cell lymphoma	Includes a humanized anti-human CD19 monoclonal antibody attached to a pyrrolobenzodiazepine dimer toxin via a valine-alanine linker
12	Tivdak	Tisotumab vedotin	TF	IgG1	Recurrent or metastatic cervical cancer in adult patients with tumor growth after chemotherapy (r/mCC)	Fully human monoclonal antibody conjugated to MMAE (targeting TF)
13	Elahere	Mirvetuximab soravtansin	FRα	IgG1	Treatment of adult patients with FRα expression-positive, platinum-resistant epithelial ovarian, fallopian tube, or primary peritoneal cancer who have received 1–3 prior systemic regimens	Using an FRα-targeting antibody, a microtubule inhibitor was phased through a cleavable linker

**FIGURE 1:**
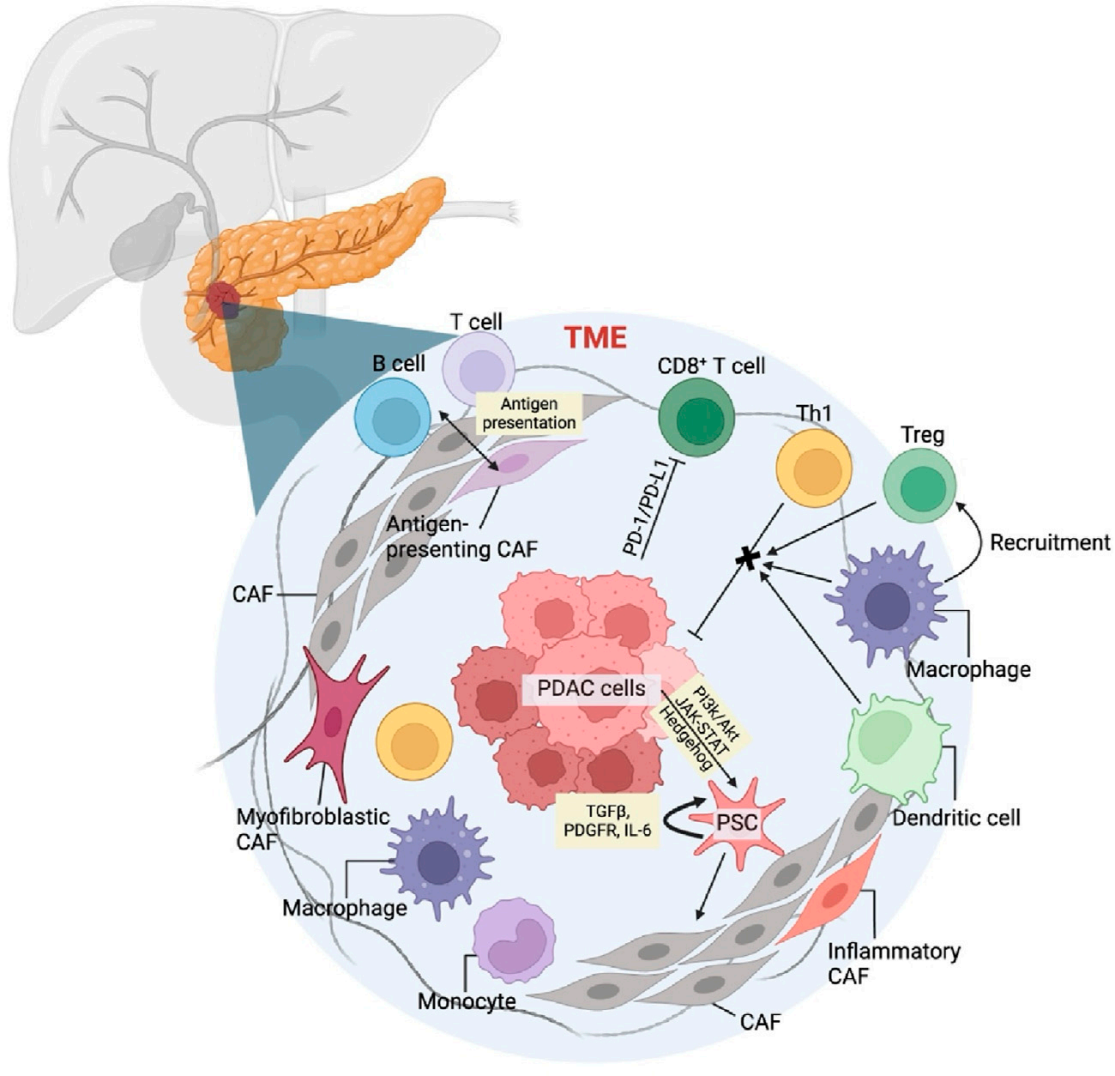
Pancreatic cancer tumor microenvironment and mechanism of TME cellular components in pancreatic cancer.

The tumor microenvironment (TME) is a complex mixture formed by the interaction of new tumor cells, stromal cells, cell matrix, growth regulators and other cellular components, which promotes the proliferation and migration of tumor cells. The tumor microenvironment of pancreatic cancer is essentially a dense matrix formed by excessive fibrosis caused by active connective tissue proliferation and deposition (Erkan et al., 2012). It has important and complex impacts on the biological behavior of pancreatic cancer (Figure 1). During the occurrence and development of tumors, a microenvironment with high rate of tumor survival, proliferation and distant metastasis is formed, which can reduce the killing effect of tumor cells and escape immune surveillance.

Cancer-associated fibroblast (CAF) is one of the hallmarks of TME which originate from primitive fibroblasts, bone marrow-derived cells, and stellate cells within the matrix. Among these, pancreatic stellate cells (PSCs) are the primary source of CAFs in pancreatic cancer (Veenstra et al., 2018). Cancer cells activate PSCs through pathways such as PI3k/Akt, JAK-STAT, and Hedgehog, as well as through inflammatory cytokines and reactive oxygen species. During this activation, vitamin A lipid droplets in the PSC cytoplasm disappear, and a significant amount of α-smooth muscle actin is produced. Activated PSCs then secrete factors like transforming growth factor β (TGFβ) (Ahn et al., 2018), platelet-derived growth factor receptor (PDGFR), and interleukin-6 (IL-6), which enhance their activity and lead to a myofibroblast-like phenotype, becoming CAFs. CAFs secrete growth factors, cytokines, and matrix metalloproteinases that promote cancer cell growth and migration (Lafaro and Melstrom, 2019; Liang et al., 2017). Additionally, CAFs upregulate ligands for programmed death receptor 1 (PD-1) and cytotoxic T lymphocyte-associated antigen 4 (CTLA-4), inducing apoptosis of effector T lymphocytes and aiding cancer cells in immune evasion (Lafaro and Melstrom, 2019).

In the tumor microenvironment of pancreatic cancer, the infiltrating lymphocytes are mainly T lymphocytes, including CD4^+^ helper T lymphocytes (Th), regulatory T cells (Treg) and CD8+T lymphocytes. CD4+Th cells can be further divided into anti-tumor Th1 and pro-tumor Th2. Despite the abundance of T lymphocytes in the pancreatic cancer stroma, the infiltration and activation of anti-tumor effector T cells are significantly hindered. Programmed death receptor 1 (PD-1) is present on the surface of these lymphocytes. When their receptors recognize the major histocompatibility complex on cancer cells, T cells become activated and secrete interferon γ (IFNγ). High local concentrations of IFNγ and tumor necrosis factor α (TNFα), secreted by tumor-associated macrophages, prompt cancer cells to express programmed death ligand 1 (PD-L1). The binding of PD-L1 to PD-1 triggers apoptosis in T lymphocytes, enabling cancer cells to evade the immune response (Boussiotis, 2016; Tsukamoto et al., 2019).

Tumor-associated macrophages (TAMs) are the most abundant immune cells in the TME and play a crucial role in promoting tumor growth and metastasis through complex autocrine and paracrine pathways. Based on their activation type and function in the TME, TAMs are primarily classified into M1 and M2 types. M1 macrophages can directly mediate cytotoxicity to exert anti-tumor effects and secrete a variety of pro-inflammatory factors and adhesion molecules to promote inflammatory responses, thereby indirectly combating tumors. In contrast, M2 TAMs secrete numerous anti-inflammatory factors and immunosuppressive ligands, such as programmed death receptor 1 ligand (PD-L1), which inhibit the immune response and foster tumor growth and progression through chronic inflammation and matrix remodeling (Habtezion et al., 2016). TAMs can switch between M1 and M2 types under different stimuli. This plasticity provides a theoretical basis for precisely targeting and reshaping TAM functions, guiding them to transition from M2 to M1, and inhibiting pancreatic tumor growth and metastasis. It is well known that macrophage colony-stimulating factor 1 (CSF1) plays a significant regulatory role in the differentiation, polarization, and chemotaxis of macrophages.

Pancreatic cancer has one of the most complicated TMEs among all the type of tumors. Common *in vitro* cell line culture often neglects the interaction of tumor cells with tumor matrix especially considering the tumor heterogeneity and an immunosuppressive desmoplastic tumor stroma environment. Many researchers have focused on the development of pancreatic cancer organoids to serve as more appropriate preclinical research models to bridge the gap between *in vitro* and *in vivo* models. Current pancreatic cancer organoids can be classified into two categories in accordance with the use of patient-derived xenograft (PDX) or not. These approaches rely on the direct collection of patient biopsies after the surgical removal of pancreatic tumors. Then one approach directly uses the collected specimen to form patient derived organoids (PDO) after certain treatment such as enzymatic and mechanical dissociation. The other approach mainly includes the tumor inoculation collected from patients to PDX. After *in vivo* development of tumors either orthotopically or subcutaneously, the desired tumors can be collected, treated and cultured as organoids together with the addition of specific factors (Sereti et al., 2023b).

The first example to describe pancreatic cancer organoids was the direct isolation of pancreatic ducts from mice pancreas in 2013, followed by Matrigel culture using AdDMEM/F12 optimized medium and suitable supplements (Huch et al., 2013). Two years later the first human pancreatic cancer organoid was reported. These organoids can be passaged indefinitely and cryopreserved, which already showed complex morphology with differing degrees of dysplastic tall columnar cells resembling low-grade pancreatic intraepithelial neoplasia (PanINs). Further constructed *in vivo* models in *Nu/Nu* mice by orthotopic transplantation also exhibited infiltrative features including poorly defined and invasive glands. The researchers then used these models to identify upregulated genes related to PDAC (Boj et al., 2015). In 2018, researchers developed an air-liquid interface (ALI) method to propagate PDOs from human biopsies and mouse tumors, maintaining the original tumor epithelium along with embedded immune cells. Single-cell analysis showed the tumor-infiltrating lymphocytes (TILs) in these PDOs accurately preserved the T cell receptor (TCR) repertoire of the primary tumors. Importantly, the human and murine PDOs successfully modeled the effects of immune checkpoint blockade (Neal et al., 2018). Another group created fused pancreatic cancer organoids (FPCOs) by co-culturing patient-derived tumors with mesenchymal and vascular endothelial cells derived from human induced pluripotent stem cells (hiPSCs) using ALI. The FPCOs were able to recapitulate two distinct aspects of PDAC tissue - quiescent, drug-resistant organoids containing abundant extracellular matrix from cancer-associated fibroblasts, and proliferative organoids that could re-proliferate after drug treatment (Takeuchi et al., 2023). Zhuolong Zhou et al. developed T cell-incorporated pancreatic tumor organoid generated by two-step packaging capable of recapitulating immune infiltration in the TME, which can mimic the mechanical barrier and allow T cell infiltration and cytotoxicity studies (Zhou et al., 2023). Daheui Choi et al. developed microfluidic PDOs similar to gold-standard Matrigel organoids with spheroid uniformity using limited cell numbers without Matrigel (Choi et al., 2024). By using these organoids, it could be much more efficient and realistic to investigate genetic cooperation, transcriptional and proteomic analyses as well as diagnostic and drug discovery, which holds promise in advance diagnosis and personalized medicine applications.

## 2 The rise of targeted therapies

Though all therapeutic regimens and drug delivery methods have side effects to varying degrees, targeting chemotherapeutic agents specifically to pancreatic cancer cells can significantly reduce toxicity and enhance therapeutic efficacy. This can be achieved by recognizing receptors on the surface of cancer cells. Key targets include Epidermal growth factor receptor (EGFR), urokinase plasminogen activator receptor (uPAR), transferrin, the cell membrane receptor ErbB2, and stem cell markers such as epithelial cell adhesion molecule (EpCAM), CD44, and CD133.

**FIGURE 2:**
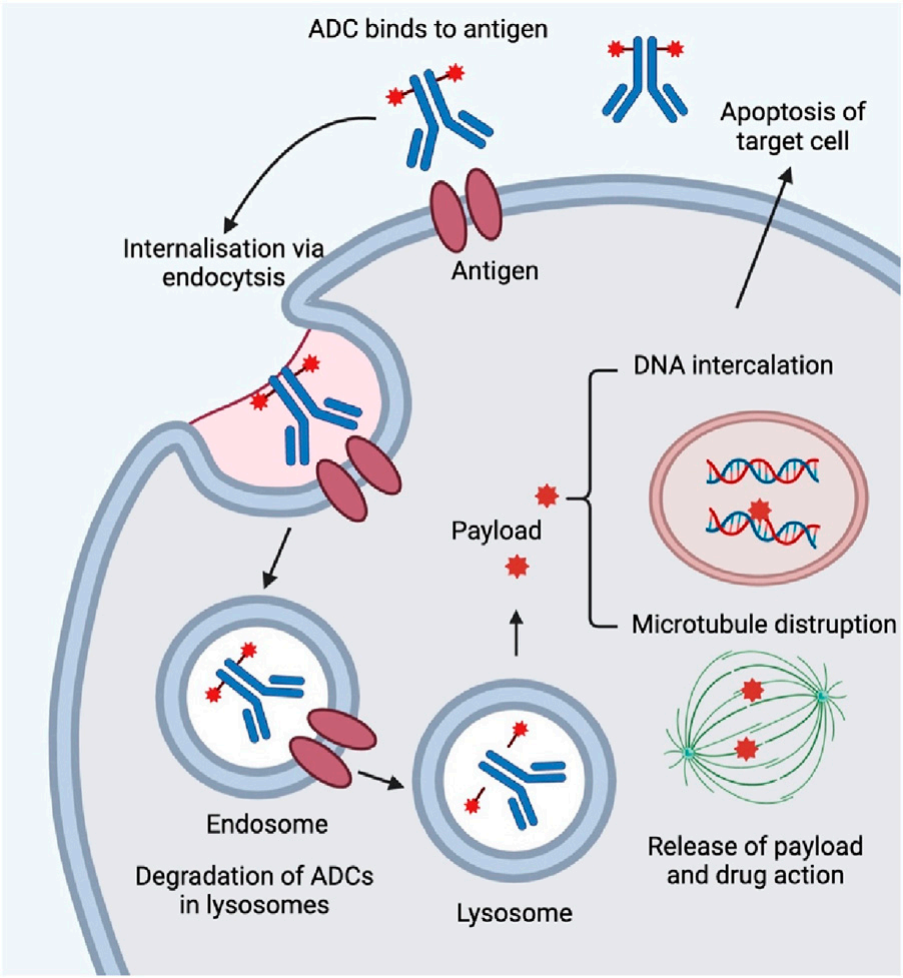
Mechanism of action of ADCs. ADCs enter human body by intravenous injection and are transported through the blood and lymphatic systems. Monoclonal antibodies bind specifically to target cells by recognizing cell-surface targets. The ADC-antigen complex enters the cell by clathrin- or caveolin-protein-mediated endocytosis and is encapsulated in the endosome. The endosome is then fused to the lysosome. The endosome is then fused to the lysosome. The early endosome matures into a late endosome before fusion with the lysosome. For ADCs with cleavable linkers, cleavage (e.g., hydrolysis, proteolytic cleavage, or reductive cleavage) occurs in either the early or late endosome. After fusion of the endosome with the lysosome, the ADCs are completely degraded by histone B and fibrillinase undergoes a complex and complete degradation by proteases. Payload is released by lysosomes into the cytoplasm to induce apoptosis by DNA insertion and inhibition of microtubule synthesis. When the target cells are apoptotic, the active payload may also kill surrounding tumor cells.

ADCs have emerged as a promising therapeutic approach in the evolving field of cancer treatment. ADCs offer a new approach to targeted therapy by combining the cytotoxicity of a drug with the selectivity of a monoclonal antibody. ADCs combine monoclonal antibody targeting with small molecule chemotherapeutic agents for targeted therapies. ADCs consist of three components: an antibody that is specialized in recognizing an antigen on the surface of a cancer cell, a payload that kills the cancer cell, and a linker to connect the two. They work by antibodies recognizing and binding to specific antigens on the surface of cancer cells, triggering endocytosis, followed by the release of a cytotoxic payload in the lysosome, leading to cancer cell death. Designing ideal ADCs requires precise selection of target antigens and design of linkers (Figure 2). ADCs need to be stable while circulating in the body to minimize off-target toxicity, while at the same time efficiently releasing the drug once it reaches the target cell. The field has grown significantly since the first ADC was approved in 2000, with a dramatic increase in the types of ADCs approved, especially since 2019. ApDCs also present as one novel targeting strategy during the preclinical study and there is also one review paper highlighting this (Dai et al., 2022). Compared with ADCs, ApDCs have advantages such as a smaller size of 6–30 kDa, preferential penetration, reduced toxicity, lower immunogenicity, and longer shelf life.

### 2.1 EGFR

Most patients with pancreatic cancer will have overexpression of EGFR. EGFR activation can trigger a series of signal cascades in cancer cells, such as cell proliferation, apoptosis, metastasis, sensitivity, and tumor vascular proliferation. This suggests that EGFR-targeted therapy may be an effective way to treat pancreatic cancer. In clinical trials using a combination of gemcitabine and the EGFR inhibitor erlotinib, survival rates improved in patients with pancreatic cancer (Moore et al., 2007). However, severe toxicities and chemotherapy side effects greatly limit the acceptability of this type of therapy. To these problems, a new EGFR-targeted ADC was synthesized, which is expected to become an effective, selective and safe therapeutic agent for EGFR-positive pancreatic cancer (Z. Li et al., 2019).

### 2.2 uPAR

The urokinase fibrinolytic source activator receptor (uPAR) is highly expressed in most pancreatic cancers, which allows it to serve as an optimal surface molecule for targeted therapies in pancreatic cancer. uPAR-targeted drugs can selectively kill uPAR-expressing cancer cells (Yang et al., 2009). In addition, the uPAR-targeted optical imaging probes were developed and used in a mouse model of pancreatic cancer, where the nanoparticles were targeted for delivery and the imaging probes selectively aggregated at the edge of the tumor, allowing residual tumors to be detected by optical imaging (Yang et al., 2013).

### 2.3 ErbB2

EGFR is part of the erbB/human epidermal growth factor receptor family of tyrosine kinases, which also includes erbB2/HER2, erbB3/HER3, and erbB4/HER4. Overexpression of EGFR is found in up to 90% of pancreatic tumors (Rabia et al., 2023). R. Ghasemi et al. explored the dual targeting of ErbB-2 and ErbB-3 in the treatment of pancreatic cancer. Using cells with ErbB-3 knockdown and a combination of EV20 and trastuzumab, they observed that this dual targeting blocked ErbB-3 signaling and inhibited cell proliferation (Ghasemi et al., 2014).

### 2.4 Tumor stem cell markers

Genetic parsing of a variety of tumor stem cells has revealed that tumor stem cells contain specific markers, such as CD24, CD44, CD133 and EpCAM, whose importance for the maintenance and activity of tumor stem cells is unclear (Vaz et al., 2013). A recent study by Tianqi Xu et al. demonstrated that the use of the monoclonal antibody Ec1-LoPE to concurrently target EpCAM improved therapeutic efficacy compared to the drug alone (Xu et al., 2023). CD44^+^ cells persist and proliferate in recurrent tumors when PDAC becomes resistant to chemotherapeutic agents Maria Inés Molejon et al. designed an experiment for recurrent PDAC in which targeting CD44 surface antigen eliminated the remaining tumor cells *in vivo* after gemcitabine treatment in a PDAC-derived xenograft model (Molejon et al., 2015).

## 3 The microscopic approaches: EM in TME analysis

EM is a very popular technique to study the biological morphology and understand the ultrastructural aspects of biological processes (Varsano and Wolf, 2022). On the basis of working principles, EM can be divided into scanning EM (SEM) and transmission EM (TEM). SEM builds up an image by sampling contiguous sub-volumes near the surface of the specimen, a fine electron beam selectively excites each sub-volume and then the intensity of some resulting signal is measured, which is used to examine dimensional topography and the distribution of the surface exposed features (Fischer et al., 2024; Joy and Pawley, 1992). Compared to SEM, TEM has higher resolution and it is usually used to observe the internal structure and details of samples, such as characterization of the newly synthesized nanoparticulates (Malatesta, 2016). In addition, volume electron microscopy (vEM) is a set of high-resolution imaging technique including serial section TEM (ssTEM), serial section electron tomography (ssET), serial blockface SEM (SBF-SEM) and focused ion beam SEM (FIB-SEM), which used in biomedical research to reveal the 3D structure of cells, tissues and small model organisms at nanometre resolution (Peddie et al., 2022). vEM techniques have been successfully applied in various fields, such as connectomics research (Briggman and Bock, 2012) virology (Baena et al., 2021) and cell biology (Narayan and Subramaniam, 2015). With technological advances in EM, the accuracy of disease diagnosis has increased. Due to its high magnification and high resolution, EM has become a popular tool for studying the structural morphology, intracellular organization and TME of various cancers including pancreatic cancer.

**FIGURE 3:**
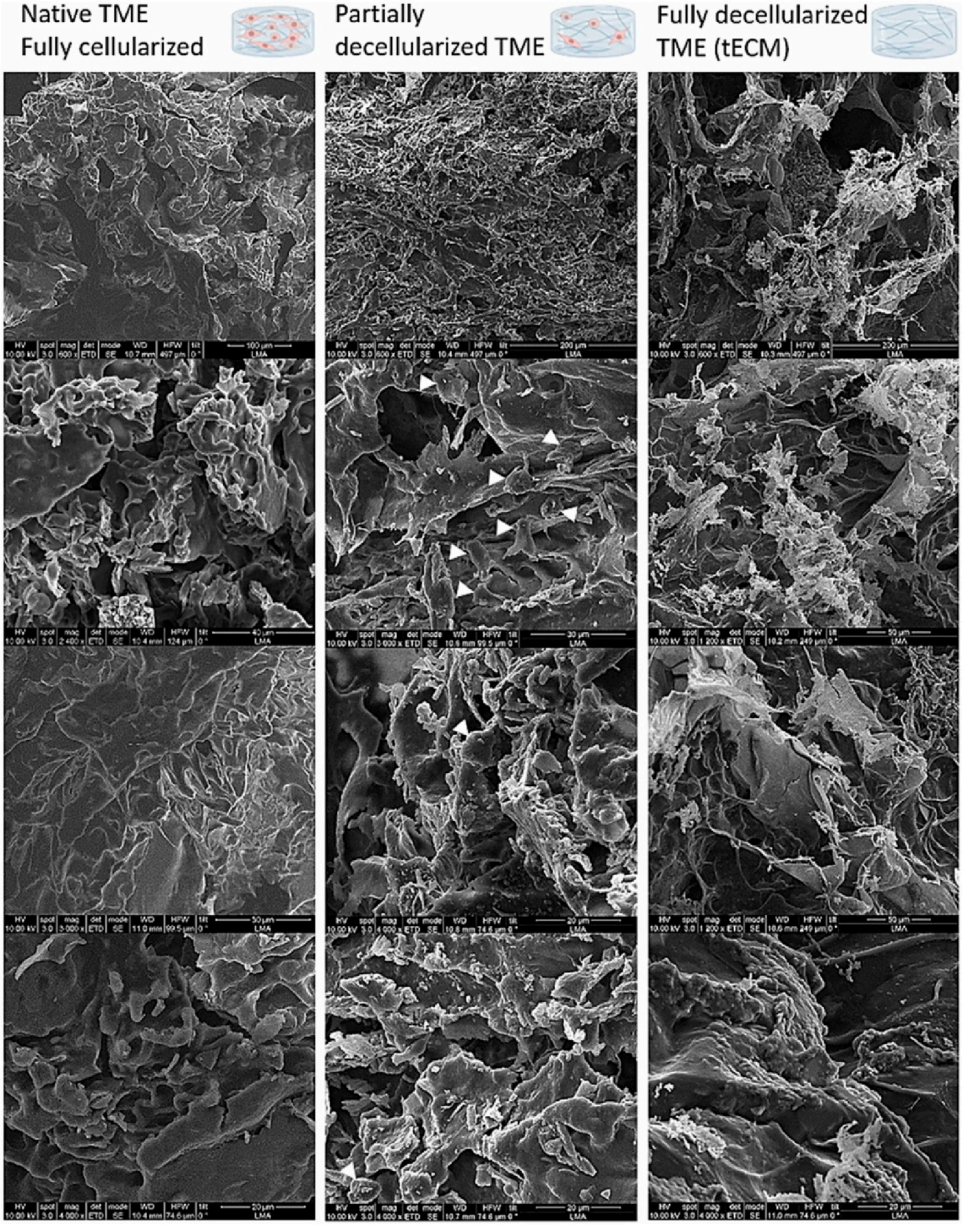
SEM images for physico-chemical characterization of the TME using one PDX PDAC-185. White arrows point at cellular remains. TME has a porous structure, aligning with earlier histological staining results. Cells form a thick, continuous layer over the tissue-engineered extracellular matrix (tECM), creating a cohesive network. As cells are removed, residual stresses are released, revealing distinct features of the tECM, such as fibers and additional pores. Cellular remnants can be observed in the partially decellularized TME group, with white arrows indicating specific instances (Garcia-Gareta et al., 2024).

The morphology of pancreatic cancer TME can be visualized with the aid of TEM and SEM. For example, PANC-1 cells exhibited a grape-like appearance, accommodating cancer cells with smooth or rough surfaces by SEM analysis and varying surface types within the spheres could be detected by TEM (Ishiwata et al., 2018). Another case is a three-dimensional pancreatic cancer model SUIT-58. SEM and TEM revealed densely congested microvillus formations at the lumen of cell spheres with junctional complexes at the intercellular part, and massive microvilli was observed in the lumen of the glands at the periphery of the organoids, similar to absorptive-like epithelium (Takahashi et al., 2021). Elena García-Gareta et al. characterized the physico-chemical features of the TME of PDAC-185 (one type of PDX) (Figure 3) and concluded TME presented an interconnected porous architecture with very low permeability and small pores due to the contractility of the cellular components (Garcia-Gareta et al., 2024). SEM are most widely-used analysis in these constructed models since it can provide a direct illustration of the shape (Sensi et al., 2023a). In addition, EM can be used as supplements for pathological identification, drug development and precise medicine.

### 3.1 Pathological identification

EM can also be used for the study of cellular mechanisms associated with pancreatic cancer cells as well as for the characterization of 3D cell lineage-like organs of pancreatic cancer. Sara Noorani et al. investigated the generation of 2.5D organoid-derived cell lines and 3D cell lineage-like organs from PDAC organoids and they observed the proliferation and migration of pancreatic cancer cells from the organoids by EM (Noorani et al., 2022).

Pancreatic cancer is difficult to cure and still lacks effective treatment options especially the most common type PDAC, due to its high incidence of distant metastasis and recurrence (Ishiwata et al., 2018). Pancreatic cancer is well-known for its poor diagnosis and detection. PDAC cell lines can form spheres (Gou et al., 2007) that colonize into a spherical shape, which was once has examined by Ishiwata et al., in 2018 using the Phenom proX desktop scanning electron microscope (Ishiwata et al., 2018). Sphere formation reveals cell proliferation under nonadherent conditions and due to this feature, enables us to detect cancer stem cells. Thus, EM provides a way to visualize and gain insights into the microscopic morphology and structure of pancreatic cancer cells, also enables researchers to investigate the cellular and subcellular changes under the condition of exposure to therapeutic substances.

As some tumors have specific ultrastructural features, for example, neuroendocrine tumors’ (NETs) can secrete granules of different numbers, sizes and ultrastructures. EM can assist in pathological diagnosis by determining the shape and characteristics of tumor cells with high resolution. Toshiyuki Ishiwata (Ishiwata et al., 2018) et al. showed that PDAC cells can be observed to form spheroids in non-adherent cultures by SEM (Gaviraghi et al., 2011; Gou et al., 2007; Ishiwata et al., 2018; Wu et al., 2011; Yin et al., 2011), which suggests that the spheroid forming cells are more likely to form a tumor than those that do not (Table 2).

**TABLE 2:** Current treatment of pancreatic cancer partially assisted by EM.

No.	Brand name	Common name	Under EM	Drug effect	Indications	Drug structure
1	Abraxane	Paclitaxel Albumin-stabilized Nanoparticle Formulation		- Antineoplastic activity - Prevent depolymerization of microtubules	- Used with Gemcitabine as the first treatment - Metastatic	Paclitaxel contained in nanoparticles (very tiny particles of protein)
2	Afinitor Votubia Zortress	Everolimus	Increased autophagosome formation^(Tai et al., 2012)^	- Immunosuppressant and anti-angiogenic - Inhibit mTOR activation - Inhibition of T lymphocyte activation	Progressive neuroendocrine tumors that cannot be removed by surgery, locally advanced, metastasized	A derivative of the natural macrocyclic lactone sirolimus
3	Xeloda	Capecitabine		- Antimetabolite - Interfere DNA, RNA, and proteins	- Adults who have had surgery removal and relapse prevention	
4	Gemzar Infugem	Gemcitabine	- Severe perturbations of the plasma and nuclear membranes - Pro-apoptotic effect^(Papa et al., 2012)^	- Antimetabolite - Disrupt the ability of the cells to make DNA and proteins	- Used alone in whose cancer is stage II/ stage III, cannot be removed by surgery or stage IV - Already treated with fluorouracil	
5	Tarceva	Erlotinib Hydrochloride		- Antineoplastic - Compete with ATP, inhibit EGFR phosphorylation	Used with gemcitabine in patients whose cancer cannot be removed by surgery or has spread	Quinazoline derivative
6	Carac	Fluorouracil		- Antimetabolite fluoropyrimidine analog - Inhibits thymidylate synthase, resulting in the depletion of (TTP)		Topical/injection
7	Onivyde	Irinotecan Sucrosofate		- Antineoplastic - Inhibition of topoisomerase I	- Metastatic - Used with oxaliplatin, fluorouracil, leucovorin calcium as the first treatment - Used with fluorouracil and leucovorin calcium in patients whose cancer got worse after gemcitabine treatment	Provided as the hydrochloride trihydrate form, which is encapsulated and entrapped within liposomes
8	Lynparza	Olaparib		- PARP inhibition - Antineoplastic	Maintenance therapy in adults with metastatic cancer that has not progressed after first-line therapy with platinum chemotherapy and has certain germline mutations in the BRCA1 or BRCA2 gene	
9	Jelmyto Mitosol	Mitomycin	Necrotic features such as cell swelling, low cytoplasmic electrondensity, and ballooning of the mitochondria^(Saito et al., 2012)^	- Antineoplastic antibiotic - Produce oxygen radicals, DNA alkylation, and interstrand DNA cross-link	Pancreatic adenocarcinoma that is locally advanced or has metastasized	Antibiotic (isolated from the bacterium *Streptomyces Caespitosus* and other *Streptomyces* bacterial species)
10	Sutent	Sunitinib Malate		Indolinone-based tyrosine kinase inhibitor with antineoplastic activity	Progressive neuroendocrine tumors that cannot be removed by surgery or metastatic	Orally bioavailable malate salt
11	Welireg	Belzutifan		Inhibitor of hypoxia inducible factor (HIF)-2alpha (HIF-2a), with potential antineoplastic activity	Pancreatic neuroendocrine tumors	

### 3.2 Drug development

EM is also a popular tool to study the delivery of targeted drugs and the interactions between drugs and cells in the drug discovery phase. For example, Evan S. Glazer MD (Glazer, Massey, Zhu and Curley, 2010) et al. observed the delivery of targeted gold nanoparticles in a study of the effects of non-invasive radiofrequency fields following treatment of pancreatic cancer with targeted gold nanoparticles, confirming the delivery of the nanoparticles to PANC-1 cells rather than Cama-1 cells were delivered to PANC-1 cells instead of Cama-1 cells by transmission EM (TEM). Additionally, Jameel Ahmad Khan (Khan et al., 2011) et al. performed the design of nano-targeted drugs against pancreatic cancer and confirmed the uptake and localization of gold nanoparticles within the cells by TEM. After the same nanoconjugates studied treated the cells, it was possible to observe whether the nanoconjugates showed up in the vesicular structure of the cells or at the periphery of the cell membrane via TEM.

### 3.3 Precise medicine

In addition, by studying the cellular structure of individual cases, EM can help physicians visualize the entire course of the disease and create more individualized treatment plans. Gabriel- Valeriu Miracia (Mirancea et al., 2014) et al. used EM to observe abnormalities in key cellular structures and cell-cell interactions in insulinoma cases, including the proliferation of β-tumor cells and differentiation between the two phenotypes, alterations in the cellular matrix and intercellular junctions, and endocellular abnormalities, matrix and intercellular junctions alterations and intracellular membrane abnormalities. They emphasized the importance of understanding these changes to prevent possible complications and improved therapeutic strategies. Through this in-depth biological analysis, medical professionals are able to better understand the complexity of insulinomas, which can lead to more effective and personalized treatment options for patients.

## 4 EM-Driven discoveries in therapeutic efficacy

### 4.1 Apoptotic revelations: the role of eicosapentaenoic acid

Eicosapentaenoic acid (EPA), as a common omega-3 fatty acid, showed its ability to induce apoptosis in human breast cancer cells in research done by Kang et al., in 2010 (Kang et al., 2010). It is reported that EPA can trigger ROS accumulation and subsequently induce caspase-8-dependent apoptosis in human breast cancer cells both *in vitro* and *in vivo* using mice cells.

In 2013, Masayuki Fukui et al. used both *in vitro* and *in vivo* models to investigate the anticancer mechanism of EPA (Fukui et al., 2013). Two commonly used human pancreatic cancer cell lines, MIA PaCa-2 and Capan-2 cells, were used as *in vitro* models. Apart from similar observations as the previously mentioned research, it was interesting to find that the pancreas could accumulate EPA at a level that was surprisingly higher than several other tissues. Therefore, it was suggested that EPA could suppress the growth of MIA PaCa-2 human pancreatic in nude mice by inducing apoptosis via specifically triggering the apoptosis-initiating proteases caspase-8 (Heimli et al., 2002). Although the ultrastructural changes in the pancreatic cancer cell were not confirmed by EM in this study, it considered that EPA could be the potential and widely accepted treatment of pancreatic cancer. There are also other researches focusing on the investigation of the anticancer effect of EPA, particularly in pancreatic cell studies, aiming at finding out the detailed and precise molecular mechanisms which include suppression of neoplastic transformation, inhibition of cell cycle (Bartram et al., 1993), enhancement of apoptosis, and antiangiogenic effect (Dekoj et al., 2007).

In 2005, Shirota et al. gave some evidence of morphological changes of the ultrastructure of the human pancreatic cell by TEM. In this study, three human pancreatic cancer cell lines (SW 1990, AsPC-1, and PANC-1) to examine the activity of apoptosis under the effect of EPA (Shirota et al., 2005). While there was another proposed EPA-induced apoptosis occurring in caspase-3–independent mechanism (Puertollano et al., 2003) which was different from the previously mentioned articles, the author suggested that the differences might be due to the different phenotype or genotype of the cell lines used. Further investigations are needed.

The method used to prepare cell investigations in this study under electron microscope followed the procedure in the article (Middleton et al., 1992) published by Middleton et al. Eventually, the study gave some clear images showing the stages of apoptosis and all consistent results were observed in 3 cell lines treated with EPA. In control PANC-1 cells, the chromatin was floccular and dispersed throughout nuclei evenly and the nuclear membrane was intact shown in the micrograph. In PANC-1 cells treated with 500 M EPA for 48 h, these early morphological changes of apoptosis were shown, which included chromatin condensation and margination under the nuclear envelope with damage to the nuclear membrane. PANC-1 cells in the later stage of apoptosis demonstrated nuclear condensation and fragmentation with the formation of apoptotic bodies. The observations match with the stages of apoptosis. Another paper published by Dini et al. has observed the morphological changes of apoptosis of U973 cells under a Philips CM12 electron microscope (Dini et al., 1996). Chromatin was initially enclosed in the nucleus. When there are substances inducing apoptosis, the chromatin starts to condense and form tiny clumps around the nucleus, which leads to nuclear fragmentation subsequently. On the other hand, Morphologic changes suggesting necrosis, characterized by cell swelling and membrane breakdown, were not observed in any of the cell lines exposed to EPA.

Another study done by Li et al., in 2005 shows similar observations. The research team used PC12 cell lines to incubate with 200 µM EPA for 72 h and examined with a Hitachi 600-II electron microscope (Li et al., 2005). Typical morphological changes of apoptosis in different stages after exposure to EPA are shown. Micrographs generated by the electron microscope demonstrated that the chromatin was fragmented and accumulated to the inside of nucleolus membrane in the early stage. In the final stage, the cell membrane wrapped up the fragmented chromatin.

### 4.2 Novel compounds under the microscope: the case of gramicidin A

Gramicidin A, an ionophore antibiotic derived from the bacterium *Bacillus* brevis1 (Dubos, 1939; Hotchkiss and Dubos, 1940), is a channel-forming polypeptide and can disrupt cellular ionic homeostasis, leading to cell dysfunction and death (David and Rajasekaran, 2015; Kelkar and Chattopadhyay, 2007; Wang et al., 2011). It acts on cell membrane by inserting one of its intermediates (Wallace, 1986), which then alter the channel membrane conformation.

Several studies have demonstrated the effectiveness of Gramicidin A against cancer cells. Its helical structure allows it to span the cell membrane. When dimerized, Gramicidin forms a pore that permits the free diffusion of water and cations, resulting in Na + influx, K+ efflux, membrane depolarization, osmotic swelling, and ultimately cell lysis. Previous research has shown that Gramicidin can inhibit the proliferation of cancer cells, such as RBE and HuCCT1 (Gong et al., 2020) and SGC-7901 gastric cancer cells and HepG2 liver cancer cells (Chen et al., 2019) by inducing apoptosis. Additionally, its effects on ovarian cancer cells include disrupting DNA fragmentation and interrupting the cell cycle, which also leads to apoptosis (Choi et al., 2023).

The structure and mechanism of the effects of Gramicidin A was commonly investigated using Atomic Force Microscope, x-ray diffraction, dissection microscope, which gives the surface structures. However, due to its high toxicity towards mammalian cancer cells, Gramicidin A has the potential value to be an anticancer agent. It is important to know the therapeutic effect of this antibiotic and its mechanism specific to pancreatic cancer cells to evaluate the possibility for it to become one of the treatment drugs. Therefore, it is crucial to examine the interaction between Gramicidin A and pancreatic cancer cells at the molecular level.

Research done by Wang et al., in 2019 shows that Gramicidin A has demonstrated satisfying effects on pancreatic cancer stem cells (Wang et al., 2019). It is used to compare with the know treatment of Pancreatic cancer, ionophore antibiotic salinomycin (Sal) which the effects have confirmed and widely accepted (He et al., 2013). With the higher resolution brought by electron microscope, conformational changes of cells including external morphological changes and ultrastructural changes of cells has been observed under the effect of this antibiotic.

Wang et al. proposed that the microvilli-like protrusions decreased significantly in both pancreatic cell Lines after exposure to gramicidin A. Normally, many microvilli-like protrusions line the cell membrane surface in both types of cells, appearing bent and thin in shape. However, as shown in the higher magnification images generated by scanning electron microscope, GrA-treated cells lost almost all of these microvilli-like protrusions. The difference of surface morphological changes of BxPC-3 and MIA PaCa-2 cells treated with 0.05 μM GrA or Sal were confirmed by micrographs. This indicates a remarkable effect of GrA on the ultrastructural changes in the membrane of pancreatic cancer cells.

Additionally, researchers Wang et al. also demonstrated how Gramicidin A works and show effects on cancer cells under the observation using Transmission electron microscope. Unlike SEM, which provides information on the cell’s surface and its composition, TEM offers more detailed insights into the cell’s inner structure by allowing electrons to pass through the sample (Ma et al., 2006). In cells treated with 0.05 μM Gramicidin A (GrA) or Salinomycin (Sal), swelling of mitochondria and disappearance of cristae were observed. These effects were more obvious in MIA PaCa-2 cells, which exhibited engulfed vesicles which contains atypical mitochondria while mitochondria in BxPC-3 cells didn’t show much aberrant observations. The results indicate that GrA has stronger effects on mitochondrial functional proteins than Sal at equal molar concentrations.

Another research done by Makino et al., in 2011 examined the 15 mer peptide antibiotic, Gramicidin A using cryo transmission electron microscope (Cryo-TEM), providing a clear structure of how Gramicidin A works on cell membranes. They form vesicles and Gramicidin A blocks are tightly packed in the membrane region (Makino and Kimura, 2011). This might be the insight for further therapeutic applications.

With the help of TEM and SEM together, researchers can examine internal and external structures of the cancer cells while investigating the therapeutic effects of responses to different drugs. Research done by Takagi et al., in 2008 compared the findings using light microscopy, confocal laser scan microscopy, and transmission EM and how they differentiate from each other’s. They were examining the microvessel density of a pancreatic, cancerous tumor. The results, though, did not directly demonstrate that the electron microscope has played a significant role in contributing to their research questions, but pointed out that TEM can identify aggregations of single spots as individual cells even when they are smaller than 1 μm (Takagi et al., 2008), whereas light microscopy and confocal laser scan microscopy only allows a lower magnification.

To sum up, Gramicidin A has great potential on cancer treatment. With the help of more advanced EM techniques, it is possible to know about the more detailed molecular mechanisms of how Gramicidin A have influences on cancer therapy.

## 5 Structure strategy against pancreatic cancer

Many researchers focus on the changes of TME in pancreatic cancer on the microscopic level by EM to understand the mechanism of the pancreatic cancer. EM is widely used in the observation of TME in pancreatic cancer due to its relatively higher resolution. It was invented by Ernst Ruska in the 1930s (Shampo and Kyle, 1997) and experienced a long period of technological innovation on sample preparation. One of the most important is the improvement ultramicrotome (Porter and Blum, 1953; Sjostrand, 1953) with glass (Latta and Hartmann, 1950) and diamond knives (Fernandez-Moran, 1953), which makes the thickness of samples suitable for EM imaging acquisition. The basic workflow of sample preparation for ultrastructural observation is starting from chemical fixation, going through staining, dehydration, embedding, polymerization and ending by sectioning, which is the major method for the identification of the ultrastructure of TME in pancreatic cancer.

With advancements in sample preparation, imaging data collection, and analysis techniques, both the ultrastructure of key regions or organelles in pancreatic cancer and the factors leading to these ultrastructural changes have gradually been revealed. For instance, tube-forming growth is a key histological feature of PDAC and relies on the activation of transforming growth factor β (TGF-β) signaling (Yamaguchi et al., 2019). Yamaguchi et al. used EM to observe the ultrastructure of the PDAC cell line, YamaPaca cells, induced by TGF-β signaling activation. EM images showed that YamaPaca-6.12 cells formed spheres with tethered cell–cell structures and a cystic lumen coated with microvilli. However, when treated with TGF-β, YamaPaca-6.12 cells developed a more loosely arranged space and tiny microvilli, indicating that TGF-β signaling is crucial for tube-forming growth (Yamaguchi et al., 2019). Additionally, Vinciguerra et al. discovered that the transcription factor Fos-related antigen-2 (Fra-2) could promote tumor progression via the IGF1 receptor in PDAC with downmodulated miR-15a by inhibiting autophagy (Rampioni Vinciguerra et al., 2024). Rui-Qi Wang’s research shows more vesicles with ultrastructure compatible with autophagic vacuoles under nutrient deprivation in control, miR-15a overexpressing, and Fra-2 silenced cells compared to their counterparts grown in nutrient-rich conditions. IGF1 treatment significantly reduced autophagosomes in control cells, but not in miR-15a overexpressing and Fra-2 silenced AsPC-1 cells (Rampioni Vinciguerra et al., 2024).

**FIGURE 4:**
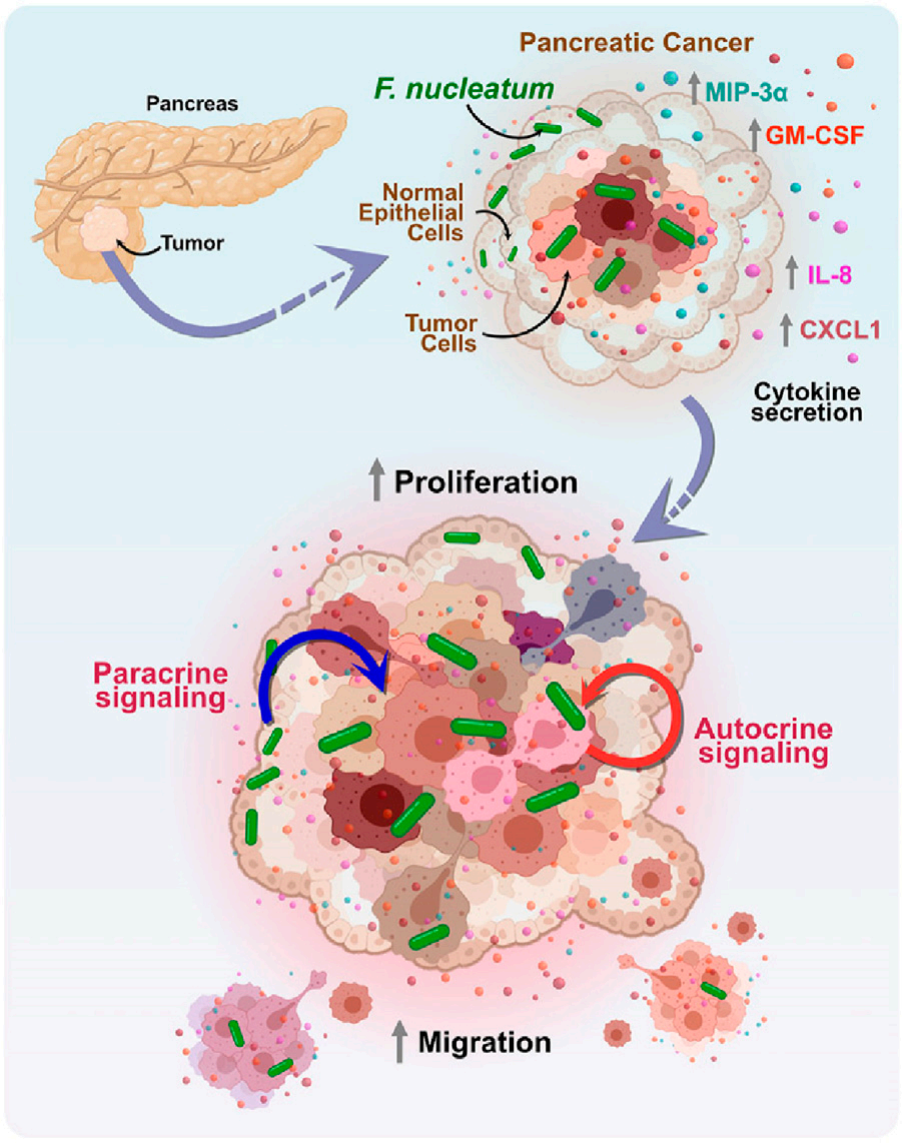
The mechanism of the effect of *Fusobacterium nucleatum* (*F. nucleatum*) on the tumor microenvironment in pancreatic cancer. *F. nucleatum* regulates the paracrine and autocrine signaling pathways between tumor cells and surrounding non-tumor epithelial cells by increasing the secretion of cytokines MIP-3α, GM-CSF, IL-8, and CXCL1. This cell-to-cell interaction promotes the proliferation and migration of tumor cells, thereby exacerbating tumor progression (Udayasuryan et al., 2022).

On the other hand, the development of EM is helpful to provide the effective drugs or methods of therapy for pancreatic cancer. Induction of apoptotic cell death is a significant method to inhibit pancreatic cancer. In 2005, eicosapentaenoic acid (EPA) was found that could inhibit human pancreatic cancer cell growth due at least in part to the induction of apoptotic cell death and observed the ultrastructure changes of nuclear membrane and chromatin between EPA treatment cells and the control ones (T. Shirota et al., 2005), in 2013, Calix [6]arene (CLX6) was characterized that could inhibit pancreatic cancer with similar mechanism above as well as induction of cell death by reticulum stress identifying by EM (Pelizzaro-Rocha et al., 2013), in 2016, *pseudomonas aeruginosa*-mannose-sensitive hemagglutinin was proved that could induce apoptosis and inhibit tumor growth (Cheng et al., 2016) and in 2017, verteporfin was found that could induces cell apoptosis in pancreatic cancer cells (R. Q. Wang et al., 2019). EM plays important roles on the above studies for its function of visualization. In addition, Ruiqi Wang’s research shows that ionophore antibiotic gramicidin A was identified as a pancreatic cancer inhibitor, which could lead to the decrease of microvilli-like protrusions on cell surface membrane by SEM and the swelling of mitochondria by transmission EM (Figure 4) (Wang et al., 2019).

Additionally, EM is a valuable tool for studying the relationship between the tumor microbiome and tumor migration. Previous research has identified positive associations between certain microbes and the development of PDAC, including *F. nucleatum* (Mitsuhashi et al., 2015). *F. nucleatum* is a Gram-negative, anaerobic, rod-shaped bacterium typically found in the oral cavity (Brennan and Garrett, 2019) and is increasingly recognized as an oncomicrobe due to its disproportionate presence in several cancers, including pancreatic cancer (Gaiser et al., 2019; Mitsuhashi et al., 2015). By using scanning EM, Barath Udayasuryan et al. found that F. nucleatum invaded BxPC3 pancreatic cancer cells, and a model of pancreatic cancer response to *F. nucleatum* infection induced cytokine signaling was developed using complementary techniques (Udayasuryan et al., 2022). Understanding how bacteria in tumors influence cancer growth and migration can lead to more effective chemotherapy methods or immunotherapies and contribute to the development of diagnostic tools and early detection strategies.

## 6 EM in the development of targeted therapies

During the production of ADCs, EM techniques were used to monitor the formation of nano-aggregates, the consistency of drug loading, etc. Ping Huang et al. used TEM to observe the morphology and size of self-assembled spherical nanoparticle aggregates during the development of an ADDC drug, which was used to compare with simulation experimental results (Huang et al., 2014). Che-Ming Jack Hu et al. used SEM to observe spherical images of targeted lipid-polymer mixing while synthesizing an anticancer embryonic antigen (CEA) hemi body conjugated to a lipid polymer targeting pancreatic cancer (Hu et al., 2010). Cary D. Austin et al. observed the surface and internal distribution of trastuzumab in cancer cells by EM, which was used to study trastuzumab and ErbB2 in the recycling pathway and the lysosomal pathway (Chalouni and Doll, 2018). Prathap Kumar Mahalingaiah et al. while investigating the potential mechanism of target independent uptake and toxicity of antibody-drug conjugates using to EM observed that Caveolar endocytosis is characterized by a spike-like coating of cholesterol related caveoli (Mahalingaiah et al., 2019). Also, Caveolar endocytosis involves the formation of a complex lattice of intercalated layers and choroidal endosomes that can also be recognized morphologically by EM.

Overall EM plays a major role in the development of ADCs to observe the drug structure as well as to study the drug release. As an imaging technology that can be accurate to the nanometer level, EM provides an important perspective for studying the behavior of nanocarriers such as liposomes and nanoparticles during drug loading and drug release. EM provides a microscopic point of view for the study of ADCs and ApDCs to observe the interactions between the drug and the antibody or peptide directly, helping researchers to better understand the structure of these complex drugs and their behavior *in vivo*. and antibody or peptide interactions, helping researchers to better understand the structure of these complex drugs and their behavior *in vivo*.

## 7 Conclusion

Although the direct application of EM in the clinical treatment of pancreatic cancer is limited, it has an important role in basic pathologic research, pathological diagnosis, and drug development of pancreatic cancer. Through EM, researchers can directly observe the microscopic morphology of cancer cells at different times as well as the tumor microenvironment, evading growth inhibitory factors, resisting cell death, maintaining proliferation signals, causing replication death, activating invasion and metastasis, and inducing angiogenesis. When conducting targeted drug and ADCs development, EM can be used to directly observe the delivery of targeted drugs, as well as the pathways of drug transportation, and the morphology of nanoparticles. Some researchers have also studied cellular structures through EM, observing changes in connections between cells and changes within cell membranes, so that individualized therapeutic strategies can be targeted or complications can be prevented. Current studies have shown that 3D scanning EM can rapidly and accurately image ultrastructures (Cohen Hyams et al., 2020). Matthia A. Karreman et al. combined 3D EM with intravital microscopy (IVM) and micro-X-ray computed tomography, which was used to obtain high-resolution 3D information on tumor cells, and this combination offers the possibility of studying these cells at higher resolution and *in vivo* (Karreman et al., 2016). The 3D electron microscope has also been used to study cellular structures in a variety of ways. ultrastructure. One of the most valuable next steps for EM in cancer research is the ability to obtain not only high-resolution 3D EM data, but also more information about how tumors develop their hallmarks over time, such as activation of invasion and metastasis.
