# Erratum: Insights and therapeutic advances in pancreatic cancer: the role of electron microscopy in decoding the tumor microenvironment

**DOI:** 10.3389/fcell.2025.1605997

**Published:** 2025-04-15

**Authors:** Frontiers Production Office

**Affiliations:** Frontiers Media SA, Lausanne, Switzerland

**Keywords:** pancreatic cancer, tumor microenvironment, EM, antibody-drug conjugates, aptamer-drug conjugates

Due to a production error, Yuying Wang was not included as an author in the published article. The corrected author list appears below. The publisher apologizes for this mistake.

“Hong Dai^1†^, Xingxuan Chen^2†^, Jiawen Yang^3†^, Yuying Wang^2^, Rodrigo Azevedo Loiola^4^, Aiping Lu^2^* and Kenneth C. P. Cheung^2^*^‡^”

The original version of this article has been updated.

